# Self-Quantification Systems to Support Physical Activity: From Theory to Implementation Principles

**DOI:** 10.3390/ijerph17249350

**Published:** 2020-12-14

**Authors:** Paul Dulaud, Ines Di Loreto, Denis Mottet

**Affiliations:** 1 Tech-CICO (Technologies for Cooperation, Interaction, and Knowledge, in Collectives), Université de Technologie de Troyes, 12 Rue Marie Curie, 10000 Troyes, France; paul.dulaud@utt.fr; 2 Euromov Digital Health in Motion, Université de Montpellier, IMT Mines Alès, 700 av. Pic St Loup, 34090 Montpellier, France; denis.mottet@umontpellier.fr

**Keywords:** quantified self, health, physical activity, behavior change, model, support system, persuasive design, user-centered design

## Abstract

Since the emergence of the quantified self movement, users aim at health behavior change, but only those who are sufficiently motivated and competent with the tools will succeed. Our literature review shows that theoretical models for quantified self exist but they are too abstract to guide the design of effective user support systems. Here, we propose principles linking theory and implementation to arrive at a hierarchical model for an adaptable and personalized self-quantification system for physical activity support. We show that such a modeling approach should include a multi-factors user model (activity, context, personality, motivation), a hierarchy of multiple time scales (week, day, hour), and a multi-criteria decision analysis (user activity preference, user measured activity, external parameters). This theoretical groundwork, which should facilitate the design of more effective solutions, has now to be validated by further empirical research.

## 1. Introduction

The quantified self movement has raised in 2007 with enthusiastic users of self-tracking devices, and the wearable device market has grown exponentially since then [1,2,3]. Self-tracking tools are now widespread among people who wish to monitor all types of information about themselves (e.g., heart rate, steps, sleep) in order to potentially change their lifestyle for better health [4].

On the scientific side, a major descriptive effort has been carried out since 2010 to define this quantified self movement, showing that the most popular objective is behavior change. Researchers agree that self-reflection and contextual factors are indispensable to achieve behavior change [5,6,7,8]. Self-reflection means that a user needs to understand his or her habits and their variations within his/her environment through collected data. The user’s environment is referred to as contextual factors or parameters: e.g., weather or schedule are parameters that influence the user’s context. Consequently, a self-quantification system must be customizable and adaptable to the user’s life to really help people change their behavior [5,6,7,8,9].

However, most current self-quantification systems are too generic. In the case of physical activity monitoring, for example, most collected data like user’s steps and heart rate provides some vague personalized tips to users such as “try to walk more”. Thus, significant commitment in data collection, management, and analysis is required from users, in order to achieve a proper understanding of their habits. Today, the steps that quantified selfers go through in a self-quantification experience have been characterized (e.g., collecting data, displaying it in a meaningful way), and guidelines for more effective self-quantification systems for behavior change have been identified (e.g., need for context, holistic approach). Nevertheless, established descriptive models and guidelines are not sufficiently precise to guide the implementation of more effective and user-adaptive self-quantification systems. It is therefore essential to derive implementation principles from the established theoretical framework and associated guidelines.

In this respect, we propose a minimal user model fitting in with the existing conceptual models. Its four components bring together the guidelines from previous research: traditional activity tracker data are aggregated into activity data (heart rate, number of steps, etc.), external data to assess the user’s context is called contextual factors (weather, schedule, etc.), user personality traits provide basic levers to customize the user experience, and user motivation accounts for the level of exercise adherence. Our model of a *self-quantification system for physical activity support* tries to bridge the existing gap between a well-defined quantified self conceptual framework and limited implementation principles. Our model is structured around the aforementioned multi-factors user model (activity data, contextual factors, personality, and motivation), a hierarchy of multiple time scales (week and day to account for human activity patterns, hour for user monitoring and feedback), and a multi-criteria decision analysis approach (user activity preferences, number of steps toward the goal, weather) for physical activity recommendations. A system built on our model should be, by design, capable of assisting a quantified selfer to understand and change his or her physical activity.

The paper is organized as follows: we begin by providing background on the quantified self movement and the main descriptive axes that have emerged from research during the past decade. Next, we detail our model for an adaptive and personalized self-quantification system for physical activity support. Lastly, we discuss some challenges that arise when designing and developing such a self-quantification system based on our model.

## 2. Characterization of the Quantified Self Movement

The quantified self movement was allowed by the technological advances in electronics and computer science of the early 2000s, but the action of collecting information about oneself has a long history.

### 2.1. Self-Tracking Background

Terms such as “quantified self”, “self-tracking”, “personal analytics”, or “personal informatics” refer to systems and practices that help people collect and reflect on their personal information [6,10,11]. In this class of human–computer interaction, people collect personally relevant information for the purpose of self-reflection, gaining self-knowledge, and better understanding their own behavior [6,7]. Broadly speaking, the current “quantified self” definition refers to the community as well as the practices of self-tracking [8]. According to Lupton, it encompasses the incorporation of technology into data acquisition of daily life in terms of inputs, states, and performance to achieve self-knowledge and self-reflection [12].

Self-tracking is over two centuries old. For example, in the 18th century, Benjamin Franklin used to track the days in which he accomplished one of his 13 virtues (like sincerity, moderation, or humility) for 60 years [13]. In the 1900s, Buckminster Fuller (an architect, designer, inventor and futurist) kept a scrapbook in which he registered every 15 min of his life [14]. More recently, Nicholas Felton, a computer graphic designer, has published famous annual reports between 2005 and 2014, focusing on “*translating quotidian data into meaningful objects and experiences*” [15]. Finally, Chris Dancy is now known as the most connected human on the planet to track every single bit of his life for several years.

### 2.2. Modern Quantified Self and Personal Informatics

Behaviors recording, in the form we know today, was initiated by technophiles in Silicon Valley in the 1970s. The process of quantifying one’s life was traditionally used in behavioral psychology for clinical and research environment [8,16]. Quantifying one’s life could help diagnosis, selection of treatments, and help to monitor changes after a treatment [17]. In the late 1990s, with the democratization of computers, microelectronics, and the development of the internet, sensors have become cheaper, smaller, and information could be accessed anywhere [8]. Consequently, sensors became available to the general public, which led to practices of using technology for self-tracking known as quantified self, since 2007 [1,12]. Today, there is an active international community sharing practices through meetups (in more than 40 countries), blogging, and annual conferences [8].

Health tracking has rapidly developed as an emerging paradigm for health care self-management [18]. Health tracking is facilitated by wearable sensors that enable the general public to easily capture health data on a daily basis [19,20]. Nowadays, health tracking technologies have overall proven to be effective on increasing awareness and behavior change [8,12].

### 2.3. Goals of Quantified Selfers

Before going any further, we need to understand what are the quantified selfers’ goals. Quantified selfers’ goals may relate to the self-management of chronic diseases [21], to general personal informatics [6], or to tracking health as a preventive tool [22].

The goals can be divided into three categories (see Table 1) [8]. Improving health includes prevention, monitoring the impact of a treatment (e.g., cardiac arrhythmia medications in a case of tachycardia), managing a particular condition (e.g., glycemic control through diet), or answering specific questions (e.g., what factors make one feel energetic in the morning). Improving various aspects of life includes, for example, determining when one is most productive or managing a budget to maximize savings. Finding new life experiences includes anything that does not have a specific goal, such as discovering new tools, learning interesting things, or having fun. Finally, people sometimes have no particular objective when starting self-tracking and want to figure out what goals would be appropriate to pursue. These people use self-quantification tools to determine whether they have a problem and what actions could fix it [6].

In this paper, we focus on the most represented category of health objectives, namely those related to activity. Physical activity comes first in the health category: activity (40%), food (31%), weight (29%), sleep (25%), and mood (13%) [8]. Activity tracking is usually associated with health risk prevention, which translates into a final objective of changing health behavior [19,20].

Quantified selfers’ goals have been identified and described precisely by previous research, but what barriers do they face in implementing a system to achieve their objective?

### 2.4. Barriers and Limits

Previous research identified the limitations in self-quantification experiences that prevent quantified selfers from successful outcomes, and proposed guidelines for system design to overcome these barriers.

From a general personal science perspective, Wolf and De Groot mentioned three barriers [1]: individuals often tinker with their own tools because of underdeveloped methods, personal science outcomes depend on commercial trackers which are unsuitable to answer individuals questions, learning requires social support that people translate into a lack of contextualization.

Human senses and subjectivity are the raison d’être of technology in quantified self. Pure self-reflection is often flawed: people have limited memory, cannot directly perceive heart rate, and may not have the time to manually count steps throughout the day, for instance. Reflecting by using memory alone makes it difficult to see patterns and trends, especially over long period of time. People may also not have the expertise or knowledge to make the correct conclusions about their observations [6]. On this basis, Choe and Li highlight limitation factors on the human side: “lack of time”, “insufficient motivation”, and “difficulty in data integration and interpretation” [6,8].

Choe and Li also highlight limitations regarding the tools used: “unsuitable visualization and analytics tools” and “fragmented data scattered across multiple platforms”. Vizer and colleagues similarly underline these barriers inherent to the tool, and an article from Epstein even reports that some people find the commercial self-quantification tools useless [5,23]. Finally, from a general perspective, Almalki and colleagues highlight that achieving a useful health outcome is pretty difficult in terms of managing data and reflecting on it, because it involves a systematic understanding of the tools and a complex undertaking of user activities [19].

More generally, a systematic approach for conceptualizing and mapping essential activities undertaken by quantified selfers is very desirable, and this is especially important because there is no comprehensive list of problems that users could experience with personal informatics [6]. The most common pitfalls among quantified selfers’ practices are “tracking too many things”, “not tracking triggers and context”, and “lack of scientific rigor” [8]. The authors also mention open questions that are inherent barriers to a self-quantification experience: how to easily explore data? How to bring scientific rigor to the quantified self movement?

### 2.5. Conceptual Models

In order to better characterize a quantified self experience, researchers propose conceptual models [5,24,25]. Here, we review three models of personal informatics for behavior change, presented in chronological order.

#### 2.5.1. Stage-Based Model

**Figure 1:**
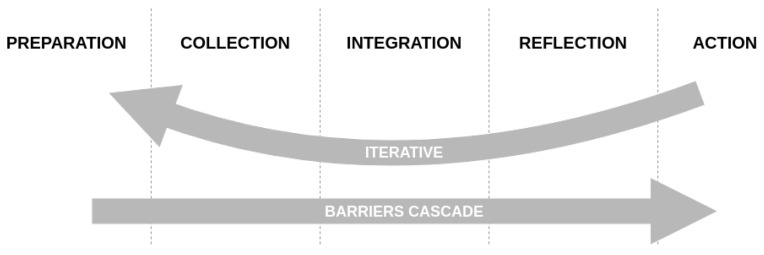
Adapted from Li et al.’s Stage-Based Model of Personal Informatics Systems: this shows the progression of a person toward behavior change through the different stages of a self-quantification experience with its iterative nature and its barriers.

The first model, the more widespread one, is Li’s stage-based model of personal informatics, which dates back to 2010 and classifies quantified selfers’ practices into five main stages in Figure 1 [6]:

The preparation stage is the very first step in a quantified self approach and occurs before information collection: people think about what information they will record and what tools they are going to use. The collection stage, as its name indicates, occurs when people collect information about themselves. This refers to the self-tracking activity from Almalki’s definitions [19]. The third step is the integration stage, where the collected information is prepared, combined, and transformed. Its duration varies a lot depending on the tools used or the information tracked and requires effort for data preparation. With the prepared data, the reflection stage starts when the users reflect on their personal information. It involves looking at the collected information or interacting with information visualization. Reflection can be short-term (makes users aware of their current status) or long-term (allows users to compare information between different times and reveals trends and patterns). Finally, the last action stage occurs when people choose what they are going to do with their newfound understanding of themselves.

The main strength of the model is the simplicity of the linear flow between the stages and the clear description of the barriers that prevent transitioning between them. Yet, this simplicity is also a weakness, because the model is not flexible and can break down when encountering the realities of everyday life [26,27].

#### 2.5.2. Lived Informatics Model

**Figure 2:**
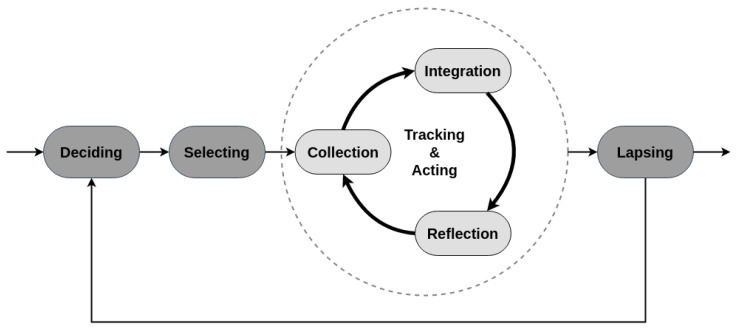
Adapted from Epstein et al.’s lived informatics model of personal informatics: this model is based on Li et al.’s model and highlights the essential fluidity and iteration of a self-quantification process. It is not specifically oriented towards behavior change, though.

In 2015, Epstein and colleagues proposed a lived informatics model (see Figure 2). The model is about general tracking in everyday life and aims to be an enhancement of Li’s model by dividing preparation stage into deciding and selecting. The model also introduces a tracking and acting cycle for iterative progression through collection, integration, and reflection. Its most interesting characteristic is that it anticipates human lapse, but it is not oriented toward behavior change only.

#### 2.5.3. Conceptual Model of Shared Health Informatics

**Figure 3:**
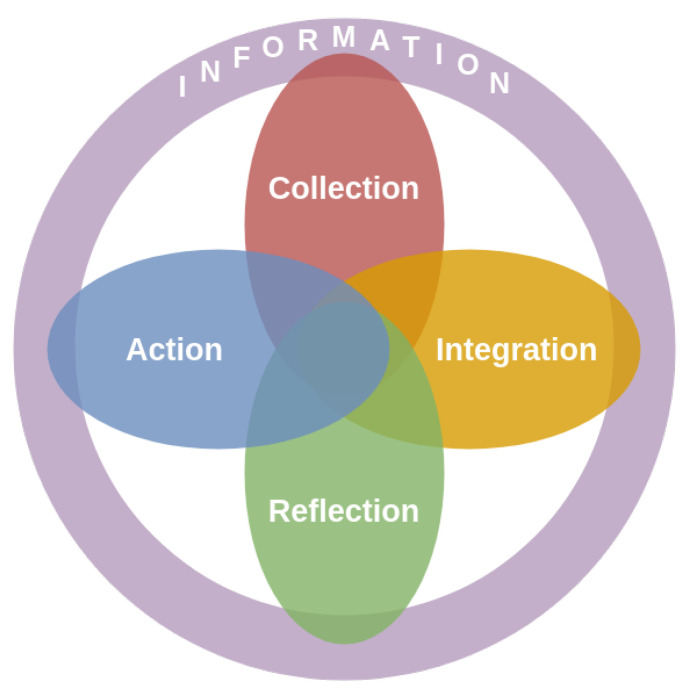
Adapted from Vizer et al.’s conceptual model of shared health informatics (CoMSHI): also based on the stage-based model, the CoMSHI enhances the fluidity of the process by facilitating transitions between stages. It reflects the need for context raised by previous research as well.

From their analysis of past literature and existing models, Vizer and colleagues have noticed a strong need for a model that more closely aligns to the unique needs of health context [5,28,29]. In the light of these observations, they propose a new model which bridges the gap between current personal informatics models and tracking for chronic illness self-management. This new conceptual model of shared health informatics (CoMSHI) is based on Li’s model, but adds communication to incorporate interactions between actors and redefines preparation to information (see Figure 3).

The main strength of the model is the unconstrained transitions between stages, which allows for different types of work to happen simultaneously, and better represents the necessary smoothness and flexibility in self-quantification experiences [26,30]. For this particular reason, the CoMSHI remains interesting for our approach, although it concerns treatment self-management of patients with chronic illness.

The three models reviewed in this section are mature and accurate enough to account for the different stages and needs occurring during a self-quantification experience and they also provide a number of guidelines (Section 2.6). However, they are of limited help when it comes to the design of more effective tools (Section 5.2, Table 3).

### 2.6. Existing Barriers and Guidelines for Design

By attempting to model the quantified self movement, researchers identified numerous barriers from which they derived guidelines for a better design of personal informatics systems. We address the most relevant ones for our approach in the present section.

#### 2.6.1. Barriers

Li et al. highlighted specific barriers for each stage of a self-quantification experience. They also explained how the barriers can chain themselves together in a cascade of barriers [6]. This aspect has been further developed by Vizer and colleagues who identified precise relations between stages [5]: not using the right tool, not collecting the right data, sparse data sets, scattered or ineffective visualizations, and difficult organization. Another barrier is that tools for health informatics do limit how we think about and design support systems, mainly because they often fall short of supporting the true range of work involved [5]. Those barriers for design are summarized in Table 2.

#### 2.6.2. Guidelines

To overcome the barriers, the most general guideline is to adopt a holistic approach, because focusing on one stage ignores the whole experience [6]. Developing a deep understanding of quantified selfers and their goals should help determining what tracking practice the tool must support. For instance, the CoMSHI model is agnostic to specific tools or data elements [5]. Li also highlights the possibility of combining multiple facets of people’s lives to enrich the value of the systems [6].

A second general guideline is that the system must be iterative and flexible by defining the necessary functionalities to facilitate transitions between types of work: as users go through the stages, they might change their mind about the tools used, about what to collect, or about collection methods [5,6]. We must consider how to empower people, so they can effectively transition between tools to track the data they need. Indeed, a single tool does not need to support all aspects of tracking work [5].

Another critical guideline emerging from identified barriers is data management. Previous research also indicates that we must select which stage should be facilitated with technology to benefit the user the most. This means applying an appropriate balance of automated technology and user control within each stage to facilitate user experience [6]. As a matter of fact, Choe et al. talk about maximizing the benefits of manual tracking which cannot be done within a fully automated system [8].

Last but not least, a personal informatics system aiming at supporting health behavior must support user’s behavior change by design. To achieve this goal, Consolvo and colleagues used various psychological theories to describe design strategies supporting behavior change. We discuss this aspect in the following section (Section 3) [31]. On the other hand, Choe indicates the need to promote self-reflection, as research has shown that reflection plays an important role in changing behaviors [8,32]. For example, early feedback can facilitate reflection and help the user to identify what to track. Froehlich et al. described different ways of designing feedback technologies [8,33].

The aforementioned guidelines are reported side by side with the previous barriers in Table 2.

## 3. Criticism of Guidelines from the Literature

As reported in the preceding section, previous research identified guidelines that are intended to provide guidance in the design of more effective self-quantification systems. However, applying such guidance to design more effective systems (e.g., with better user experience and improved outcomes in behavior change) remains a challenge in our opinion. There is a need to formalize its guidelines by adopting a point of view closer to the implementation.

Among the guidelines summarized in Table 2, only data management can be considered sufficiently low level and actionable, because data storage and format can be directly taken into account when developing a self-quantification system. On the other hand, guidelines such as “holistic approach” or “iterative and flexible system” need to be further specified to become actionable. Concerning the “holistic approach” to be adopted, some questions emerge and must be answered: how to focus on the overall experience rather than on discrete stages? How to reach a good understanding of the user and his/her objectives? How to combine different aspects of the user’s life? For instance, the CoMSHI adequately describes self-tracking for chronic illness self-management from an abstract point of view, but it does not provide any clue on how to build a self-quantification system. Similarly, for system iteration and flexibility, how can we ensure smooth transitions between the different stages? How can we design a system that is adaptable to the user?

Finally, regarding health tracking and behavior change specifically, the requirement to support user behavior change by design remains an overly vague guideline that needs to be more clearly defined. In that direction, Consolvo and colleagues [31] identify eight design strategies derived from the analysis of psychological theories (goal-setting theory [34], transtheoretical model of behavior change [35], presentation of self in everyday life [36], and cognitive dissonance theory [37]). They also used anterior persuasive technology projects such as Fish’n’Steps or Breakaway that they complemented with their own analysis [38,39]:

1. Abstract and Reflective—use data abstraction, on Li’s integration stage for example, to encourage the user to reflect on his/her behaviors.
2. Unobtrusive—collect and present data unobtrusively by limiting interruptions and making data available anytime.
3. Public—present personal data to the user in a way that s.he is comfortable with if other people see it.
4. Aesthetic—devices and displays must sustain interest, be comfortable and attractive to support the user’s personal style.
5. Positive—use positive reinforcement to encourage change, reward the user for performing the desired behavior and attaining a goal.
6. Controllable—permit the user to manipulate data so that it reflects the behavior he/she deems suitable.
7. Trending/Historical—provide information about the user’s past behavior relating to his/her goals.
8. Comprehensive—account for the range of behaviors contributing to the user’s desired lifestyle.

Although these interesting results provide a more precise look at the support by design of user behavior change, we believe that they are not all at the same level with regard to the implementation of a self-quantification system.

Hence, there is still a need for more detailed guidelines to build an adequate self-quantification system. Naturally, designers cannot take into account such guidance in the same way depending on the self-quantification system aimed at. While the unobtrusive strategy of a system can probably remain similar from one implementation to another, its iterative nature must certainly be adapted according to the behavior change aimed at it (e.g., physical activity versus sleep).

We believe that a mature approach to behavior change in computer sciences should rely on a good theoretical framework, as well as on good implementation principles. As a step towards this end, we propose, in the following section, an applicative and hierarchical model for a self-quantification system for physical activity support, which is illustrated by a use case example.

## 4. Model for a Self-Quantification System for Physical Activity Support

Research over the past decade has described the environment of a user involved in a self-quantification experience with precision. However, these models provide abstract indications that are impractical to implement a self-quantification system. In order to better design self-quantification systems, it is necessary to have applicative models. This is especially true for physical activity tracking, which is the main concern of quantified selfers.

Most commercial systems used by quantified selfers offer limited adapted experience and personalized advice [5,6,7,8,9]. For example, the Fitbit app allows users to set personal goals (e.g., daily steps) and activity reminders (e.g., every hour if 250 steps have not been taken). However, the Fitbit app does not assist in goal settings by taking into consideration the physical health status of the user. Similarly, the Fitbit app does not take into account the context, so as not to disturb with activity alerts when the user is usually inactive while at work. A more adapted approach would be to accompany the user in the evaluation of his or her physical health status, as well as in the management of his/her progress and motivation.

### 4.1. Use Case Example

Let’s imagine an IT professional, Phil, 40 years old, who spends most of his working day sitting in front of a computer. He is quite aware that inactivity is bad for health, so he forces himself to do one workout a week, on the weekends, such as a short run or walk on sunny days, but he would still like to be in better shape in order to improve his health status.

Every day, Phil drives to work in less than fifteen minutes but loses five minutes in traffic. He then parks at the bottom of the building where he works, then goes up two flights of stairs instead of using the elevator, because he knows that this is better for health. Once at his office, he cannot move a lot during the working day because his job mainly consists of computer work and meetings. Actually, the only significant activity he does during the day is for lunch break as he goes downstairs and walks to a food truck.

Phil is willing to improve his physical condition but lacks motivation, time, and most importantly, knowledge to understand how to do it. To help him with motivation, his wife gives him a new activity tracker for his birthday. For the first days, it is fun watching his daily number of steps and his heart rate. After a few weeks, he feels perplexed by the meaninglessness of the data he is presented with: indeed, his tracker wants him to walk 250 steps hourly and to reach 10,000 steps daily, whilst he is currently not even reaching half of it. In addition, he sees data on his activity, heart rate, and sleep, but there is no obvious connection between them.

With the feeling of forcing himself towards activity goals that are radically different from his current lifestyle and not adapted to his job and availability, Phil decides to try an experimental self-quantification system for physical activity support that a friend told him about. Apparently, this open source and self-hosted software is compatible with different activity trackers and, as an IT man, he is aware of the potential risks associated with personal information and health data analytics. Thus, having such a local solution suits him very well. He downloads the said software, installs it on his computer following the instructions, and downloads the associated app on his smartphone.

For the first day, Phil is asked to answer a personality questionnaire which identifies him as rather open to novelties, but more introverted than extroverted, conscientious and agreeable. He also answers several questions regarding his lifestyle and physical activity preferences. For the next few days, the system remains silent, but Phil knows from the documentation that the system is learning his habits and activity patterns by retrieving and analyzing the data from his tracker.

Then, after this first typical working week, the self-quantification system informs Phil that he is not very active on weekdays: this can be summed up as a couple of minutes’ walk and two flights of stairs in the mornings, a total of ten minutes’ walk during lunch breaks, another couple minutes of activity in the evenings after work, and some scattered steps in between. Phil also learns that he is not particularly fit (this is OK, he already knew that), with a resting heart rate around 80 bpm. His physical activity is very similar from one day to another during the working days regardless of the weather (this is new insight to him, however). On average, Phil reaches 4000 steps per day, with a peak of 6000–7000 steps on Saturday and mostly light activity on Sunday: this corresponds to a sedentary lifestyle. As he goes through the information he is provided with on this lifestyle, Phil is alarmed: he did not know quite as much about the risks associated with inactivity.

At the same time, Phil is somewhat reassured that the self-quantification system is now able to support him with personalized recommendations to help increase his physical activity levels, and that activity characterization will be continually refined. Phil learns that his general objective should be twofold: spreading physical activity over the week to achieve a more homogeneous profile, as well as increasing daily activity to reach higher levels.

To this extent, on the first Monday of the support phase, the self-quantification system estimates an optimal challenge point: last Monday, during learning phase, Phil reached 3500 steps, had slept moderately well, and the weather was pleasant. This Monday is not particularly sunny, but the system has assessed that Phil’s activity does not depend on the weather, that he had a good night sleep, and that he is also rather conscientious. Thus, the self-quantification system might set an optimal challenge point to 4200 steps with a half-day goal of 2000 to start with. Phil is pretty confident with a goal within his grasp. So, after having lunch with his colleagues, he goes out for a walk rather than going straight back to his office, which allows him to go beyond his sub-goal before returning to work. The app congratulates him by displaying his progress, and informs him that he should reach the 4200 steps smoothly by tonight. While parking at home, Phil is alerted that he is still 500 steps short, so he decides to park further to walk a little bit more on his way back.

After three weeks, Phil is still achieving his daily objectives, compensating for sub-goals failure due to unforeseen circumstances when necessary, and actually had the idea of scheduling his meetings in rooms on the upper floors to walk more at work. He is even thinking of initiating walking-meetings, which could result in free physical activity for everyone, as well as shorter and more efficient meetings. He definitely wants to test his colleagues on this point. His support system even informed him that his resting heart rate had decreased slightly, which was the beginning of an improvement in his physical condition.

However, today is Saturday and this is a rainy weekend: usually it is on sunny Saturdays that Phil is most active, because he goes out running. Even if the day’s objective has been revised downwards to take into account the context (rainy, slept quite well but moderately motivated), Phil has already missed his half-day goal. The system determines that he is likely to end the day very far from the initial objective, so the support loop is activated to offer him personalized activities classified by “adaptation to the current context”: play hide-and-seek with his children, follow a short indoor sports session, go out for a walk anyway, go for a run outside. Phil chose the first suggestion because he did not think that this could be considered as physical activity. In the end, even though he reached a lower level of activity than usual, Phil learned that an hour of hide-and-seek was equivalent to 2000 steps, which he never would have imagined. He plans to play another game to get even with his children tomorrow, which will not only allow him to spend time with them, but will also keep him active over the weekend. Finally, Phil also plans to go for a run on a weeknight when the weather is better, in order to keep his weekly workout going.

After a few months, Phil regularly achieves 6000 steps on working days, as he decided to cycle to work when it does not rain: it takes a little bit longer than driving, but he arrives relaxed and wide awake, having taken around 2500 steps. He has made good overall progress and learnt how to manage his activity: as an example, he is aware that he is going to lack some activity if it rains and drives to work instead, so he tries to compensate with indoor activities or more frequent short breaks whenever possible. Phil was also able to assess the effects of increased activity levels on his health as he now sleeps better, has lost a little weight and feels more in shape. He is even willing to set a personal target of at least 7000 steps per day in order to attain an active lifestyle.

This scenario illustrates the use we intend to make of our literature review to address the problem of genericity of current tools. We propose a hierarchical model relying on an evolutive user profile as a design basis for a self-quantification system for physical activity support. This applicative model relies on the conceptual ones previously described in Section 2.5 and follows previous research guidelines explained in Section 2.6: it aims to be flexible, adaptive, and aware of the user’s context to support him/her on a personalized basis towards his or her goal of physical activity behavior change.

### 4.2. Learning Phase

The goal of this initial phase is to learn the user’s health behavior pattern in his or her context. We want to discover the user’s physical activity patterns, health status, and habits in order to develop a deep understanding of the user. Physical activity patterns are relatively similar from week to week [40,41,42,43]. The learning phase should monitor the user for at least an entire week, by recording daily steps, heart rate, sleep, weather, etc., to be able to estimate with sufficient precision how s.he behaves in terms of physical activity in his/her particular context. For instance, the learning phase could determine if user activity is evenly distributed throughout the day, if it is more concentrated in the morning and evening in the case of a desk job, or how much it depends on the weather or sleep quality [44].

Consequently, user physical activity profile depends on a recurring weekly time scale, while user behavior change relies on adapted objectives that are based on daily and hourly time scales (see Section 4.3).

In addition, relying on personality traits helps to better individualize the support to behavior change through exercise adherence [45,46,47,48].

From the previous analysis, the learning phase establishes four parameters: 1. the user personality traits from the five-factor model questionnaire (openness, conscientiousness, extraversion, agreeableness, neuroticism), 2. a preference model of the user regarding physical activity (when it is the highest motivation for physical activity [49,50], what kind of activities are usually performed, what is the intensity of the activity), 3. the influence of the user’s context on activity (does the user have a desk job, does the weather affect activity level, how motivation affects the level of daily activity), and 4. the user’s general health status (how fit are they). This corresponds to the first three stages of Li’s model (preparation, collection, and integration) and includes Vizer and colleagues’ contextualization and fluidity aspects.

Thus, an ideal self-quantification system should have all the necessary elements to improve user self-reflection, understanding of health behavior, motivation and exercise adherence, hereby leading to behavior change.

### 4.3. Support Phase

After the initial learning phase, the system enters a support phase where the user will be supported towards the desired change in health behavior. Adding Li’s reflection and action stages, the main constraint here is adaptation, so we rely on Epstein’s and Vizer’s models to account for the required flexibility and potential user failure during the experience [5,30]. We want the user to achieve higher activity levels but, as noted by Frost and Smith, “Anyone who has tried to go on a diet or exercise plan can relate to this: It is always hard to adhere to rigorous behavior modifications” [51]. An adaptive system must therefore respect the user regarding his/her current state of mind, availability, and motivation.

To this extent, the system must propose personalized objectives to the user on a daily time scale. To do so, we use the newly acquired knowledge of the user and a multi-criteria decision analysis to determine an optimal challenge point: goal difficulty must be in line with user physical condition, motivation, preferences, context, etc. [52,53,54,55,56]. For instance, if one usually achieves around 5000 steps on Tuesdays without significant intensity and is in the following context: prefers to walk alone, more motivated than usual, and sunny weather, an adequate optimal challenge point might be to set a daily activity goal of 6000 steps, with a moderate intensity walking recommendation during lunch break if necessary.

To support a user in increasing his/her physical activity level, we articulate our model around three different time scales: a weekly time scale as a basis for user profile (corresponding to the learning phase which has been described in Section 4.2), a daily time scale used to set optimal objectives based on the previously determined user profile, and an intraday time scale (e.g., hourly) necessary to monitor user progress toward daily objective and to help him/her if necessary [9,57]. This aims to maintain a sufficient motivation to achieve an unusual level of physical activity while avoiding disengagement.

#### 4.3.1. Daily Time Scale

**Figure 4:**
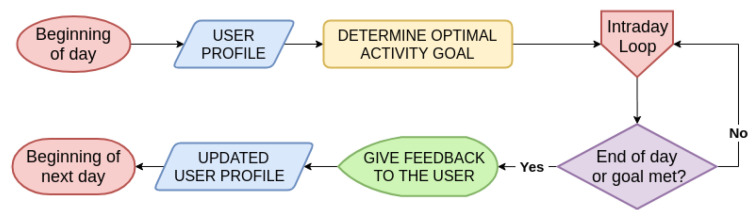
Adaptive System for Physical Activity: Support Phase—Daily Time Scale. After the initial learning phase, we know the user’s activity patterns, as well as physical health, personality, and context that compose the user profile. Hence, we are able to determine an optimal challenge point for the current user day based on his/her profile before monitoring the progress in separate intraday loops.

**Figure 5:**
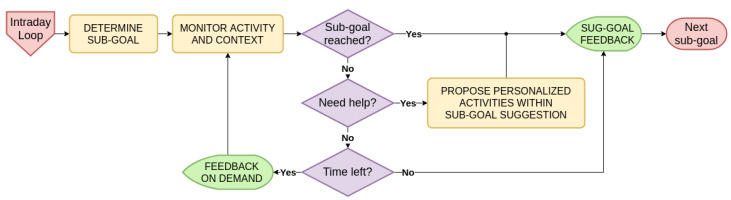
Adaptive System for Physical Activity: Support Phase-Intraday Sub-Goal Time Scale. An ideal sub-goal (3000 steps halfway through the day for example) is determined according to the objective of the day (e.g., 6000 steps). A control loop is run hourly to monitor user physical activity level and to evaluate if she is making good progress toward the sub-goal. If self-management (left loop) is not sufficient, the system can intervene to propose the user a personalized physical activity adapted to the current context (right inner loop), or move to the next sub-goal in case of failure (right outer loop).

As illustrated in the flowchart Figure 4, every day starts by using the user profile obtained after the preceding learning phase (Section 4.2) to set an optimal activity goal for the user. The optimal challenge point determination depends on activity levels achieved in the previous weeks, context (weather, schedule), user personality, and motivation. With this optimal daily objective set, our adaptive model goes down one level through intraday loops (see Figure 5), as long as the objective has not been met or the day has not ended. This allows one to continuously monitor user progress, as well as context, and to adjust accordingly if necessary.

At the end of the day, or when the goal is met, it is important to give feedback to the user regarding his/her progress. This is a behavior change technique leveraging the user’s motivation [58]. Feedback should also be used to reinforce user understanding of physical activity habits and the impact on overall activity level, sleep quality, mood, etc. The last step of this daily loop is to update the user’s profile at the end of the day given his/her performance to help adjust the optimal challenge point for the following day.

#### 4.3.2. Intraday Time Scale

Once the optimal challenge point for the day has been set, we enter intraday loops. Intraday loops allow the self-quantification system to monitor the user and his/her context. At the start of each intraday loop (e.g., hourly), the support system determines an ideal sub-goal which would give the user a roadmap to reach the optimal challenge point set previously by the end of the day. This is indeed easier to walk 200 steps five times in a day than walking 1000 steps at once in the evening. The self-quantification system is able to continuously monitor the objectives achievement rate and to adjust subsequent sub-goals according to the general objective of the day. This process is repeated until the goal is reached or there is no time left for it (flowchart Figure 5). In the intraday loops, there are three possible ways for the user: self-management, system support, or failure.

In the first case, self-management (left loop), the user’s motivation is sufficient to reach higher levels of activity on his/her own with no help nor recommendations, only by having objectives set [49,50,59]. Although this is the best-case scenario for a health behavior change, a support system still has to ensure that user motivation will not vanish in the long run.

If the user is not able to achieve a sub-goal, the self-quantification system can support (right inner loop) with a set of personalized physical activity suggestions (see Section 4.3.3 for details) which best suit the user’s preferences, current context, and current sub-goal. For instance, if a user working at a office usually prefers walking outside, a personalized and adapted physical activity in rainy weather could be to take 300 steps by going down two floors and up the stairs on the opposite side.

Finally, the worst-case scenario that must be taken into account is user failure (right outer loop), as highlighted by Epstein et al. [30]. Failure is inherent to humans, so an adaptable self-quantification system must manage this unpredictable possibility by design, because a user may experience temporary demotivation or unexpected unavailability. In such a case, the model simply moves on to the next sub-goal, which will be adapted according to the circumstances.

In any event, feedback is necessary to help the user to understand the impact of his/her actions on his/her physical activity for the day.

#### 4.3.3. Personalized and Adapted Physical Activity Choice

With the functioning and sequencing of the different time scales now explained, we further develop the description of the support loop presented above. An effective self-quantification system for physical activity support must supply its user with significant advice, personalized recommendations and proposal for context-specific activities. In this regard, we extend the multi-criteria decision analysis initiated for the optimal challenge point (cf. Section 4.3) to deal with the selection of personalized and adapted physical activities: the main question here is “how should a self-quantification system for health behavior support applied to physical activity select appropriate activities for a user”?

With the user profile previously described, we derive a user preference model for physical activities complemented by a questionnaire. This aims to identify the user’s preferred activities: if the self-quantification system detects cycling every day, occasional running, but relatively little walking, there is a good chance that our user would prefer a run rather than a longer walk.

**Figure 6:**
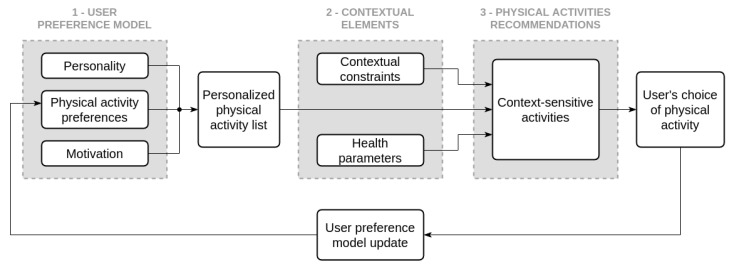
Personalized and Adapted Activities Suggestions Process: this figure details how a self-quantification system for physical activity support should rely on a user preference model of activities, (1) before filtering it with monitored contextual elements (2) in order to produce its recommendations (3). A personalized list of context-sensitive activities is proposed to the user from which they can choose.

We then filter out unsuitable activities regarding the current contextual elements weather, availability, or health parameters, in order to obtain a list of context-sensitive activities. Lastly, the initial user preference model is updated with the user’s choice to refine future suggestions as illustrated in Figure 6.

As an example, if the weather is damp for a user who enjoys cycling and running more than walking, feels drowsy because of a bad night sleep, and needs helps to reach the missing 500 steps to the half-day sub-goal; they may be presented with a choice of activity ranging from most to least adapted to the context: stretching (calm and indoor but might not reach the sub-goal so might require to be compensated for later), moderately active indoor activity (easier to meet the half-day target but physically more demanding), etc. All things considered, our user could simply choose to go for a short walk outside despite the weather because they want to get some fresh air. This choice is then logged to update the user preference model for future suggestions.

The support phase shall accompany the user during the entire self-quantification experience until higher daily activity levels become habitual. Ideally, when new activity patterns are established, the user should be able to maintain these habits without the help of a self-quantification system. We deepen this aspect in the discussion (see Section 5.4).

### 4.4. Towards an Application of the Model: System Design and Development Challenges

In the previous section, we presented what we consider to be an ideal model for a physical activity behavior change support system. Here, we discuss the main challenges of implementing our model in a self-quantification system.

First of all, our model implies an important challenge regarding its cornerstone, the multi-factors user profile: how can we mix different parameters such as personality traits, contextual variables, activity tracker data, and motivational questionnaires answers in a significant way? This challenge requires integrating several tools including a reliable personality test: we propose to use the Big Five Inventory, as it tends to be the most trusted and tested model regarding treatment acceptance [60,61]. It is also easily usable from an IT point of view. Then, relevant contextual parameters are required (APIs can regularly be used to collected weather data from public services or user availability from a connected agenda). Physiological data are naturally retrieved from an activity tracker worn by the user. Our model also requires a motivation and exercise adherence assessment tool: the literature is quite extensive in psychological research and an interesting possibility may be an “approach and avoidance” mathematical modeling that involves user input in the form of a questionnaire [50,62,63].

As we have seen, good feedback is mandatory for a self-quantification system aiming at supporting health behavior change [33,58]. This essential part should ease the user reflection regarding his/her health status and habits, hereby alleviating formatting, analyzing, and associating data with contextual elements. The main challenge is how to present efficient and meaningful feedbacks to the user. Is it feasible to use automatic statistical analysis and correlations? How can we combine context elements with statistical analysis? How can we personalize user feedback depending on the user profile? Some trails of reflection have already been explored by previous projects such as the role of feedback in the process of change, effects of immediate feedback, or using personality traits to support personalization and feedback in a sleep health behavior change support system [32,45,64]. Such research showed for example that feedback helps to reach more directly decisional consideration and to increase motivation.

In addition, a significant work has to be carried out on defining the user’s optimal challenge point to adapt daily objectives difficulty: how can we weigh up the established user profile with contextual elements to best match the user’s capacities, motivation, and availability? On this point, results from medical and psychological research can be exploited, but it would also be interesting to explore the potential links between goal-setting theory [34], optimal challenge point [52,53,54,55,56], personality, and physical activity [47].

Another implication for system design and development is the adaptability of time dimensions: our model relies on different time scales for user analysis, goal settings, recommendations, and monitoring which is fundamental for a tool adapted to humans. As a consequence, the time constant of each scale can be modified to better suit a user: depending on his/her job for example, a user may have very different availability so the sub-goal time scale can possibly shift from half-day to every two hours. The challenge is to determine on which basis the time scales can be adjusted to the user.

Because we are dealing with sensitive health data, our comprehensive approach inevitably raises security and privacy issues. Although our model cannot be inherently thought to be privacy-proof (a system can be built on using commercial tools and several servers around the world), we strongly recommend the usage of open source, local, and self-hosted tools. If the need to move to cloud computing is preponderant, it becomes critical to secure hosted health data. Relying on trusted third parties subject to European legislation would be a guarantee of a better user acceptance factor [4,65,66,67,68,69].

Finally, designing such a self-quantification system must categorize the interventions that it could perform to benefit the user. We suggest to rely on Wolf and De Groot activities for personal science [1]: an ideal system could indeed intervene to assist its user on questioning, designing, observing, reasoning, and discovering.

These reflections regarding the challenges of a system design based on our model are naturally entrusted to future research. Our goal was to provide a model that translates indirect guidelines from the quantified self theoretical framework into concrete implementation principles for physical activity behavior change. This point is expanded in the last section below (Section 5).

## 5. Contributions and Limitations of the Model

In the previous Section 4, we presented an applicative model for the design of an ideal self-quantification system to support physical activity. In this final section, we summarize our model’s framework and discuss its contributions and limitations.

### 5.1. Summary of our Model’s Framework

This article presents an original and minimal model to better inform the design of self-quantification systems for physical activity support. We identified a necessity to bridge the gap between a conceptual framework and an applicative model, the latter being necessary for an efficient system implementation. Wolf and De Groot indicate that “*translating common features […] into designs that can be easily shared and adapted for personal use by many people will lower the barrier to participation*” [1].

As a consequence, we rely on a review of the literature to understand the characteristics of the quantified self movement and to propose a complement to the existing abstract models [5,6,8,30]. Our model is hence based on quantified selfers’ goals and barriers, on existing theoretical models, and on indirect guidelines from past research that we strived to translate into implementation principles.

### 5.2. Analysis and Results: Comparison of Our Applicative Model against the Previous Conceptual Ones

The conceptual models (Section 2.5) are descriptive of the quantified self movement and cannot be mobilized as they stand to design more effective systems.

The stage-based model is well established in quantified self research and accurately reflects the different stages of an experience. Nonetheless, it remains too linear and leaves little room for flexibility. The lived informatics model accounts for the fluidity required in a self-quantification process, and focuses on the continuity of experience, but is not fully oriented toward behavior change. The conceptual model of shared health informatics emphasizes the need for context around an unrestricted process, but focuses on chronic illness management. The following table summarizes the point-by-point comparison of the models (cf. Table 3).

Our applicative model differs from the previous conceptual ones in its approach: we leverage on the models describing what to do to derive guidelines on how to do it. To make the approach concrete, we focus on physical activity as a target for health behavior change and we follow a user-centered design from the outset. From conceptual models, we grouped some stages together in interoperable phases (learning phase, support phase) with several level of intervention (three different time scales). Thus, our model makes it possible to account for a very personalized user experience with efficient support by being flexible, allowing simultaneous work, and considering possible inconsistencies due to the human factor.

### 5.3. Contribution

We identified that most current barriers to self-quantification experiences relate to the lack of principles to implement the abstract theoretical framework that previous research established. We therefore present an applicative model of a self-quantification system for physical activity support that emphasizes such implementation principles. Our model is intended to more precisely inform the development of such systems via: 1. a multi-factors user model, 2. a hierarchy of multiple time scales, 3. a multi-criteria decision analysis.

To the best of our knowledge, such a multi-factor user model mixing psychological aspects together with quantitative data has never been proposed. The need for iteration and flexibility is inherently implemented thanks to the different time scales and loops we used. A weekly scale for activity patterns, a daily scale for user objectives, and an hourly scale to monitor progress allow one to provide a high degree of adaptability. Finally, a multi-criteria decision analysis based on a user activity preference model, on measured variables, and on external parameters allows for a self-quantification system to produce personalized suggestions of adapted physical activities.

According to Khakurel, functional wearable devices must respect three specific points that we strived to satisfy throughout the design of our model [4]: mobility and augment reality are inherent to the use of activity trackers, which come in the form of connected watches supplying users with real time data about their actions, and context sensitivity is addressed by our multi-factors user model, as well as the mutli-criteria decision analysis.

Following the guidelines already set out, we also consider the user experience together with the system as a whole. This holistic approach enables our model to first focus on user understanding by analyzing activity patterns, habits, and physical data, then on tailoring an adapted support by determining optimal challenge points, sub-goals, and support loop. In that sense, the model is minimal, because removing any part from it would inevitably deviate from the holistic approach advised by previous research and would no longer allow one to build a self-quantification system for physical activity support in a personalized and adaptive way.

All things considered, in comparison with existing high-level models, we here present a generic model for learning support (or habit building from a psychological perspective). We adopted an approach centered on physical activity, but the genericity of our model enables it to be compatible with different problem classes such as sleep or diet, and with different age groups as well. This would certainly require some tuning of the parameters to the problem, but it is likely that the core algorithm can remain unchanged.

### 5.4. Limitations and Future Works

We are aware that our research suffers non-negligible limitations that require future work. We detail these drawbacks in the present section.

From an implementation point of view, it is not yet possible to assert that the model we propose significantly improves on the previous ones: there is no self-quantification system for physical activity support based on this new model. As we have shown with the comparative table (cf. Table 3), our approach seems legitimate from a theoretical point of view, but an experimental extension is definitely necessary in order to demonstrate its practical usefulness. However, running such experiments first require to develop a stable prototype, and we need to answer several questions in this regard. The multi-factors user profile development involves collecting and mixing heterogeneous data types, such as activity (number of steps, heart rate, sleep quality), contextual variables (weather, user availability), and personality trait scores. Then, we have to investigate the motivation part to settle on a exercise adherence assessment tool. The implementation of different timescales involves storing and using data based on three time constants as well as managing intrinsic interactions between the loops. Finally, data weighting needs to be addressed as well: mixing several heterogeneous data types requires, for instance, to look into the preponderance of personality in front of the weather for activity recommendations, or the preponderance of health status regarding motivation for objective settings.

Upcoming experiments will also raise the issue of the targeted user groups: system prototypes will have to answer the question of specific parameters to adapt our generic model to users’ age group, physical condition, etc. Verifying this genericity requires applying the model to the development of a system for experiments involving different age groups and different domains. In this regard, a limitation of our model is the case of a patient-therapist relationship, which requires a particular focus on specific groups as shown by Vizer, and colleagues with the inclusion of therapists in previous models [5].

The human factor is the first drawback related to the usage of the system: we cannot force a user to perform an activity if they do not want to, nor can we oblige a user to supply a system with inputs when this is required. Thus, we might not obtain all of the necessary data from the user every time that we will need to, and this is a point that must be taken into account when designing and developing a self-quantification system. User freedom, however, limits the effectiveness of any tool, hence that is not specific to our model but to all self-quantification approaches. Still related to the human factor is the technology acceptance of wearables studied by Khakurel [4]: although we are not proposing a device but a model to improve self-quantification outcomes, common reflections can be made regarding the adoption of a system based on our model. The most significant reported factors being technology, health, and privacy, a future system design will have to specifically address these aspects so as not to affect user behavior and intention of use. As an example, Khakurel reports that “*for quantified self tracking wearable devices such as activity monitoring to be useful in the long term […], the devices must be easy to use, intuitive, robust, and reliable. Deficiencies in these areas significantly reduce the users’ motivation*”. Finally, because we focused on a support model, a self-quantification system should avoid any form of user technology dependency. This is why we believe that users should be able to maintain their new habits without the help of a support system when better activity patterns are established. Despite the fact that some individuals might need continuous assistance from their support system in order to maintain healthier behaviors, the main objective of quantified self for physical activity is to become more aware of our activity. Thus, an ideal self-quantification system should act as a scaffold to help the user reaching consistently higher levels of activity and getting independent of the tool. The system should not make decisions for the user, but only make recommendations. We believe that we could avoid the risk of the tool taking precedence over the user’s thinking by creating explicit and well-phrased recommendations. For example, if we explicitly state that we are suggesting a short activity because the user only has 15 min in his/her calendar, in the long run, the user should be able to choose a short activity whenever they have some time. However, we could not translate this idea in our model, but future prototypes must study it on a case-by-case basis.

The other drawbacks are linked to the implementation of the system and primarily concern the use of the Big Five personality traits: this might be a controversial topic, as there is currently no general consensus in psychology research [70]. However, ongoing research on patient treatment acceptance or exercise adherence looks promising and can be adapted to our applicative model [45,47,61,71]. If future experiments show relevant correlations between personality traits and physical activity, motivation, exercise adherence, or data visualization, this would allow one to better tailor self-quantification systems to every single user. As a result, users would improve their ability to understand and change their behaviors [72].

The next drawback relates to motivation and behavior change. The discussion about this point is similar to the use or personality traits: in the field of psychology, several theories of behavior change are in competition without clear consensus. As an example, we can cite the transtheoretical model of behavior change from Prochaska that describes people’s different levels of motivation and ability to change behavior (used to classify people’s readiness to change behavior), Bandura’s self-efficacy theory that relies on competence alone for ensuring adherence, or Ryan’s self-determination theory, that takes into account volition and autonomy [49,73,74]. Future work must assess the most suitable theory for building a self-quantification system on. Similarly, the way we can incorporate motivational aspects in the design and development of such a system will also require complementary work.

The last one concerns the use of commercial activity trackers. This is the only point that does not respect our will of free and open-source tools: in the current market and research configurations, activity trackers usually synchronize user data on the manufacturers’ servers via their smartphone app. In the case of Fitbit, for instance, collected data are sent to servers in America without the user being informed of the operations that are carried out on it. An ideal solution would use an open source activity tracker like OpenHAK or okinesio, but these are very experimental, if not abandoned, solutions which are not mature enough for our purpose.

## 6. Conclusions

The quantified self movement is now well characterized with conceptual models and identified mechanisms. However, quantified selfers encounter many barriers in their experiences that greatly mitigates potential outcomes. Most of them use commercial tools such as Fitbit or Garmin solutions to monitor physical activity. Unfortunately, these systems remain too generic in their approach to the user: despite the impressive amount of data that they collect, personalization to the user and adaptation to his/her lifestyle is still very minimal. Consequently, only sufficiently motivated quantified selfers achieve positive outcomes from self-tracking, the rest of them facing the inherent barriers of the tools they use with limited understanding of their health habits.

Personalization of the interaction, application of motivational theories, and good understanding of one’s habits can significantly increase positive outcomes from self-quantification experiences. We thus propose a model for physical activity support that we believe will be valuable for future designs and developments because it synthesizes observation, advice, and guidelines from previous research in an applicative way.

This contribution provides the necessary theoretical groundwork for future prototypes and experiments, but validation of the model is still needed to demonstrate its practical utility. Experimental work needs to be conducted to evaluate the ease of implementation and the results of self-monitoring with our personalized and adaptable physical activity support system.

## Figures and Tables

**Table 1:** Quantified Selfers Goals Categorization, adapted from Choe et al. This table summarizes quantified selfers’ goals into three groups with relevant examples.

Improving Health	Improving Other Aspects of Life	Finding New Life Experience
- to cure or manage a condition	- to maximize work performance	- to satisfy curiosity
- to find triggers	- to be mindful	- to have fun
- to answer a specific question	- to trigger events	- to discover new tools
- to identify relationship		- to learn something interesting
- to execute a treatment plan		- suggestion from another person
- to make better health decisions		
- to find balance to improve health		

**Table 2:** System Design Barriers and Guidelines. This table summarizes the identified barriers and resulting guidelines to design an effective self-quantification system.

Barriers	Guidelines
- not using the right tool	- adopting a holistic approach
- not collecting the right data	- designing an iterative and flexible system
- sparse data sets	- facilitating data management
- ineffective visualizations	- supporting user behavior change

**Table 3:** Principal Models of Quantified Self. This table summarizes the present and absent characteristics of the main descriptive models of quantified self.

	Stage-Based Model of Personal Informatics (2010)	Lived InformaticsModel (2015)	Conceptual Model of Shared Health Informatics (2019)
**Characteristics of the model**	- Personal informatics framework. - Focused on behavior change. - Linear sequence of stages. - Barriers identification for each stage.	- Extension of the 2010 model. - Less focused on behavior change. - Circular sequence of stages. - Flexibility allowing interruption and resumption of use.	- Extension of the 2010 model. - Focused on chronic illness self-management. - Simultaneity of stages (no sequence). - Includes patient and therapists.
**Absent from the model**	- Explanation on how to account for identified barriers. - Precise guidelines for system design to account for the sequence of stages.	- Explanation on how to account for flexibility and human lapse. - Precise guidelines for system design to account for a circular sequence.	- Explanation on how to account for patient and therapists. - Precise guidelines for system design to account for simultaneous work.
